# β‐endorphin differentially contributes to food anticipatory activity in male and female mice undergoing activity‐based anorexia

**DOI:** 10.14814/phy2.14788

**Published:** 2021-03-04

**Authors:** Caitlin M. Daimon, Shane T. Hentges

**Affiliations:** ^1^ Department of Biomedical Sciences Colorado State University Fort Collins CO USA

**Keywords:** body weight, food intake, POMC, proopiomelanocortin

## Abstract

Anorexia nervosa (AN) has a lifetime prevalence of up to 4% and a high mortality rate (~5–10%), yet little is known regarding the etiology of this disease. In an attempt to fill the gaps in knowledge, activity‐based anorexia (ABA) in rodents has been a widely used model as it mimics several key features of AN including severely restricted food intake and excessive exercise. Using this model, a role for the hypothalamic proopiomelanocortin (POMC) system has been implicated in the development of ABA as *Pomc* mRNA is elevated in female rats undergoing the ABA paradigm. Since the *Pomc* gene product α‐MSH potently inhibits food intake, it could be that elevated α‐MSH might promote ABA. However, the α‐MSH receptor antagonist SHU9119 does not protect against the development of ABA. Interestingly, it has also been shown that female mice lacking the mu opioid receptor (MOR), the primary receptor activated by the *Pomc*‐gene‐derived opioid β‐endorphin, display blunted food anticipatory behavior (FAA), a key feature of ABA. Thus, we hypothesized that the elevation in *Pomc* mRNA observed during ABA may lead to increased β‐endorphin concentrations and MOR activation to promote ABA. Further, given the known sex differences in AN and ABA, we hypothesized that MORs may contribute differentially in male and female mice. Using wild‐type and MOR knockout mice of both sexes, a MOR antagonist and careful analysis of food anticipatory behavior and β‐endorphin levels, we found 1) increased *Pomc* mRNA levels in both female and male mice that underwent ABA, 2) increased β‐endorphin in female mice that underwent ABA, and 3) blunted FAA in both sexes in response to MOR genetic deletion yet blunted FAA only in males in response to MOR antagonism. The results presented provide support for both hypotheses and suggest that it may be the β‐endorphin resulting from increased *Pomc* transcription that supports the development of some features of ABA.

## Graphical abstract

Rodents with access to a running wheel and time‐restricted access to food develop activity‐based anorexia such that they run before and during food presentation, eat very little, and lose significant weight. Neurons in the hypothalamus have been implicated in the development of this anorexia‐like behavior, but there remains an incomplete understanding of the underlying neurocircuitry. The data here show a role for the hypothalamic transmitter β‐endorphin in the development of the food anticipatory behavior in male and female mice, although pharmacologic inhibition of β‐endorphin signaling only inhibited the running behavior in male mice. This sex‐specific action of the opioid‐receptor antagonist suggests that targeting this receptor in male patients with anorexia may have greater clinical value than antagonist use in females, although more work is needed to extend this work into humans.

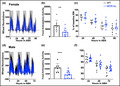

## 1 INTRODUCTION

Anorexia nervosa (AN) has a lifetime prevalence of up to 4% in women and less than 1% in men (Keski‐Rahkonen & Mustelin, 2016; Smink et al., 2012) and has a high mortality rate at roughly 5–10% (Arcelus et al., 2011). Diagnostic criteria for AN include low bodyweight, intense fear of gaining weight, and disturbed body image perception (American Psychiatric Association, 2013). While not a formal criterion for diagnosis, excessive exercise is an extremely common feature observed in AN patients, with one study reporting the behavior in over 80% of those surveyed (Casper et al., 2020; Rizk et al., 2020). Unfortunately, no prevention or early intervention strategies for AN currently exist despite an obvious need. Moreover, while current treatment therapies are often initially effective, relapse frequently occurs (Khalsa et al., 2017). Despite the severity of this disease, surprisingly little is known regarding the neurobiological basis of AN (Zipfel et al., 2015). It is crucial that the gaps in knowledge be addressed to facilitate the development of novel therapeutics for AN.

Studies using animal models have been instrumental in probing the underlying mechanisms of AN; one of the most widely used and well‐established animal models is activity‐based anorexia (ABA) (Klenotich & Dulawa, 2012). ABA closely mimics several key features of AN including severely restricted feeding and excessive exercise (Welch et al., 2018). In ABA, timed feedings restricted to limited hours of the day paired with access to a running wheel results in pronounced reductions in food intake and body weight loss as well as pronounced increases in wheel running activity. Wheel running activity is particularly increased in the hours preceding food presentation during the light cycle in which rodents are typically inactive, a phenomenon referred to food anticipatory activity (FAA; Mistlberger, 1994). Remarkably, animals will continue to lose weight and engage in wheel running to the point of exhaustion and death, as noted in the original reports describing this phenomenon over 50 years ago (Hall & Hanford, 1954; Routtenberg & Kuznesof, 1967). Sex differences have been reported in animals undergoing ABA though this difference remains incompletely understood as both sexes have been identified as more vulnerable to ABA compared to the other (females more vulnerable: Figure 1 in Klenotich & Dulawa, 2012, Pare et al. 1978; males more vulnerable: Achamrah et al. 2017; Doerries et al. 1991). Despite the evidence suggesting a sex difference is likely albeit incompletely understood, many ABA experiments previously where only conducted in one sex, usually female, given that females are diagnosed with AN in greater numbers than males (Zipfel et al., 2015).

**FIGURE 1:**
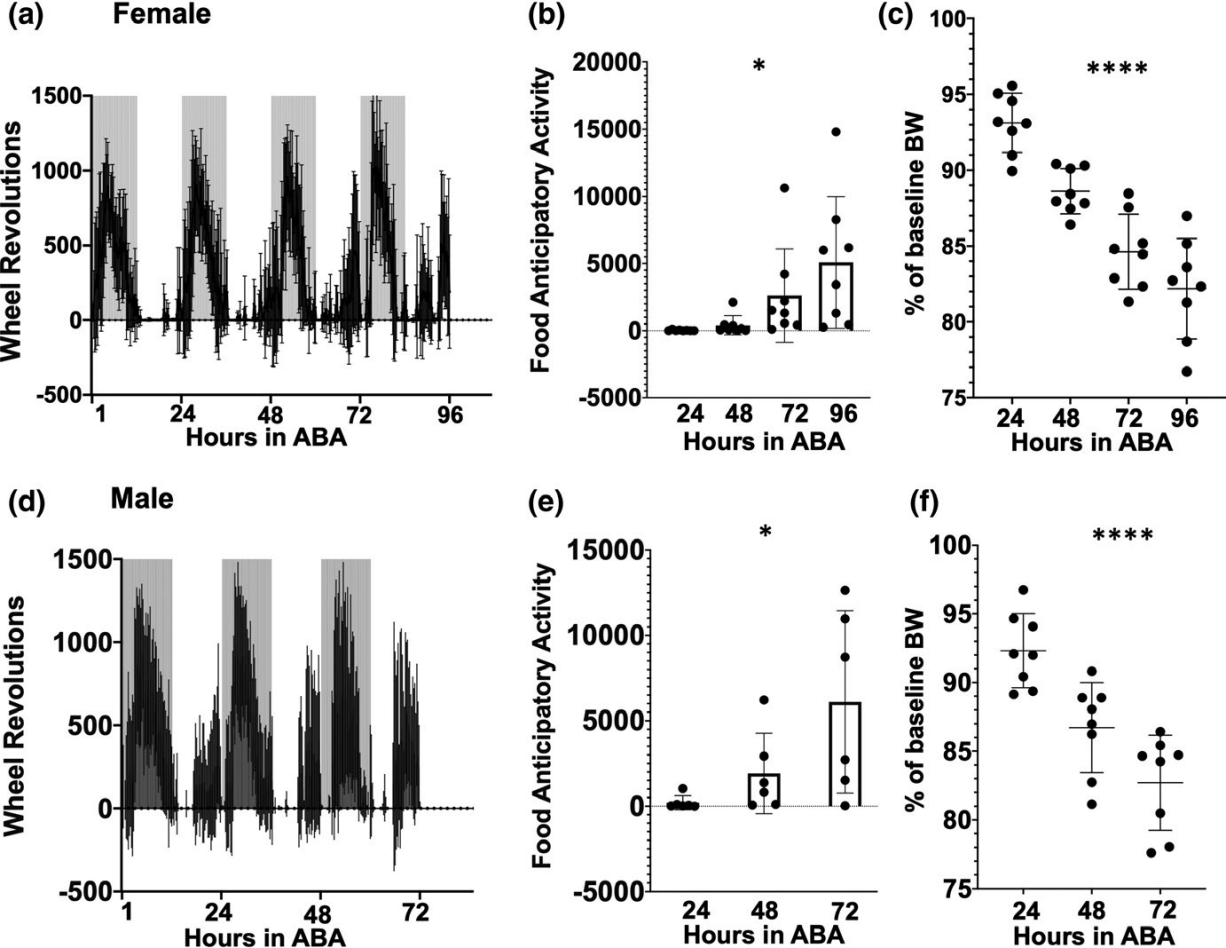
Activity‐based anorexia (ABA) can be reliably generated in wild‐type mice. Wheel revolutions per 15‐minute time bin over the course of 3 days in ABA are shown for female (a) and male (d) mice. Gray bars denote the dark cycle. Wheel revolutions during FAA, the four hours preceding food presentation, are shown in panels b (female, n = 8; *p = 0.0224) and E (male, n = 7; *p = 0.0334). Daily bodyweight expressed as a percentage of the animal's baseline average is shown in panels c and f for females and males, respectively. In both panels c and f, ****p < 0.0001. Summary data are presented as mean±SD. Data were analyzed using repeated measures one‐way ANOVA. Additional details are found in the results section

Using the ABA model, a role for the hypothalamic proopiomelanocortin (POMC) system has been implicated in the development of ABA as *Pomc* mRNA is transiently elevated in female rats undergoing ABA (Hillebrand et al., 2006). As a prohormone, POMC is enzymatically cleaved in multiple bioactive peptide products (Cawley et al., 2016; Harno et al., 2018) and previous investigations of POMC involvement in ABA have focused primarily on the cleavage product α‐melanocyte stimulating hormone (α‐MSH) given its ability to robustly inhibit feeding via activation of the melanocortin‐4 receptor (Fan et al., 1997; Huszar et al., 1997). Yet while exogenous administration of α‐MSH was found to exacerbate ABA (Hillebrand et al., 2005), subsequent experiments in which the melanocortin receptor antagonist SHU9119 was administered showed the drug incapable of ameliorating ABA (Hillebrand et al., 2006). Interestingly, it has also been shown that female mice lacking the mu opioid receptor (MOR), the primary receptor activated by the *Pomc*‐gene‐derived opioid β‐endorphin, display blunted food anticipatory behavior, a key feature of ABA (Kas et al., 2004).

In the current study, we hypothesized that the elevation in *Pomc* mRNA observed during ABA may lead to increased β‐endorphin action and MOR activation to promote ABA. Further, given that AN disproportionately affects women, we hypothesized that MORs may contribute differentially in male and female mice. We first confirm the findings reported by Hillebrand and colleagues in female rats that *Pomc* mRNA is transiently elevated in female mice undergoing ABA as well as report a similar finding in male mice. We then show that circulating levels of β‐endorphin increase in response to the ABA paradigm and that inhibiting MOR activation during ABA selectively reduces FAA but does not alter bodyweight or food intake in either male or female mice. Finally, we found a sex‐ and behavior‐specific difference between the genetic deletion of MORs and pharmacologic inhibition of these receptors. These results indicate a potential sex‐specific degree of involvement of the β‐endorphin system in ABA and suggest that there could be a need for sex‐specific approaches to treatment in patients with AN.

## 2 MATERIALS AND METHODS

### 2.1 Ethical approval

All experiments were performed in accordance with the *Guide for the Care and Use of Laboratory Animals* set forth by the National Institutes of Health and were approved by Colorado State University's Animal Care and Use Committee under protocol 19‐9730A. All experiments comply with the ethical policies of the Journal of Physiology.

### 2.2 Animals

Mice were initially acquired from the Jackson Laboratory (C57BL/6J, 000664 and B6.129S2‐*Oprm*1^tm1Kff^/J, 007559) and bred at Colorado State University. Standard PCR techniques were used to genotype animals. Male and female mice aged 2–6 months were used in all experiments. Animals were maintained on a 12/12‐hour light/dark cycle with *ad libitum* access to food and water unless stated otherwise. In the breeding room where animals were housed prior to entering an experiment, lights turned on at 06:00 hr. The experimental procedure room was on a modified light/dark cycle with lights turning on at 02:00 hr. Following transfer to the procedure room, all animals were given a minimum of 10 days to adjust to the altered light/dark cycle. During the acclimation period of ABA (discussed in detail below), wheel running activity was collected and analyzed to verify that proper adjustment to the altered light/dark cycle had occurred. All mice adjusted to the altered light/dark cycle prior to the start of the experiment. Room temperature was kept constant in both the breeding facility and the experimental test room at 20–22°C.

### 2.3 Activity‐based anorexia model

The activity‐based anorexia (ABA) model is a well validated, commonly used behavioral paradigm in which access to a running wheel paired with restricted feeding results in severe weight loss and reductions in food intake, in addition to increased wheel running activity (Klenotich & Dulawa, 2012). At the start of all experiments, mice were singly housed in clean cages equipped with a running wheel (catalog # 0297; Columbus Instruments, Columbus, OH). Mice were given 3 days to acclimate to their new environment during which Multi Device Interface Software (Columbus Instruments, Columbus, OH) detected the total number of wheel revolutions every 15 minutes. Data were collected during acclimation for two reasons: first, to confirm proper adjustment to the altered light/dark cycle; and second, to determine whether the mouse exhibits sufficient baseline wheel running activity to warrant moving forward in the experiment. Mice running less than 1500 revolutions a day were considered nonrunners and were not used for ABA. Following the acclimation period, baseline daily bodyweight and food intake values were collected 1 hour prior to lights out for 5 days. Wheel running activity continued to be monitored every 15 minutes. Following baseline data collection, the ABA paradigm or relevant control condition was initiated. Food restricted animals were given access to chow for 2 hours a day, presented at the start of the dark cycle. Males and females were always run separately, and cages and wheels were thoroughly cleaned in between cohort runs. Control conditions included mice in cages where running wheels were provided but locked in place to create a food restricted without wheel running condition (FR only) and *ad libitum* fed animals provided access to a running wheel (WHL only).

### 2.4 Assessment of the temporal dynamics of Pomc mRNA expression or β‐endorphin concentration

For determination of *Pomc* mRNA and β‐endorphin levels, male and female mice were sacrificed at lights out after varying lengths of exposure (1 day, 2 days, or 3 days) to the ABA paradigm (FR +WHL) or one of the control conditions. No differences in *Pomc* mRNA expression were detected in either control condition regardless of day of sacrifice; as such, control data were pooled. At sacrifice, animals were first deeply anesthetized with 200 mg/kg sodium pentobarbital solution (Fatal‐Plus, Vortech Pharmaceuticals, Ltd, Dearborn, MI) and lack of deep pain reflex confirmed before transcardial perfusion with 10% sucrose followed with 4% paraformaldehyde in potassium phosphate‐buffered saline. Brains were then stored at 4°C in 4% paraformaldehyde until sectioned. Whole blood was collected from the right atrium prior to perfusion and allowed to clot for 30 minutes at room temperature prior to centrifugation at 3000 rpm × 20 minutes at 4°C. Serum was removed and stored at −80°C.

### 2.5 Fluorescent in situ hybridization

Fluorescent *in situ* hybridization was used to detect *Pomc* mRNA as previously described (Dennison et al., 2016; Jarvie et al., 2017). In brief, brains stored at 4°C in 4% paraformaldehyde were sliced into coronal sections (50 μM) spanning the rostral–caudal axis of the arcuate nucleus. Slices were then incubated at room temperature sequentially in: 6% hydrogen peroxide, Proteinase K (10 μg/ml), glycine (2 mg/ml), postfixation solution containing 4% paraformaldehyde, and 0.2% glutaraldehyde and finally ascending concentrations of ethanol prior to incubation in hybridization solution for 1 hr at 60°C (66% (v/v) deionized formamide, 13% (w/v) dextran sulfate, 60 mM NaCl, 1.3x Denhardt's solution, 13 mM Tris‐HCL, pH 8.0, 1.3 mM EDTA, pH 8.0). The *Pomc* probe (0.25 pg/ml, corresponding to bases 531–1000 of GenBank sequence NM_08895.3) was denatured for 5 minutes at 85°C, added to the hybridization solution, and hybridized at 70°C for 18–20 hr. Brain slices were washed in saline sodium citrate buffer posthybridization before detection of the fluorescein isothiocyanate‐labeled *Pomc* probe with a secondary antibody conjugated to Alexa Fluor 488 (1:400, Invitrogen/Thermo Fisher Scientific, Waltham, MA). Tissue sections were mounted on glass slides, cover‐slipped, and stored at 4°C for later image collection and analysis.

### 2.6 Image collection and analysis

All images were collected on a Zeiss 800 confocal microscope at 40x. Imaging parameters were kept consistent between experiments and each experiment contained both control and experimental animals. For each animal, a minimum of 10 tiled z‐stack images taken from 10 brain slices at 1‐μm intervals were obtained containing both sides of the arcuate nucleus. *Pomc*‐expressing cells labeled with AlexaFluor‐488 were identified using masks generated in ImageJ. The fluorescent intensity of each *Pomc*‐expressing cell was expressed as a percentage of background fluorescence intensity for that given image. An overall average of fluorescent intensity above background was generated for each animal by averaging the values collected from individual z‐stack images.

### 2.7 Radioimmunoassay

Peptide extraction and β‐endorphin measurement on previously stored serum samples were performed using a commercial radioimmunoassay kit according to the manufacturer's instructions (RK‐022–33, Phoenix Pharmaceuticals, Inc., Burlingame, CA). In brief, samples were incubated overnight at 4°C with rabbit anti‐β‐endorphin antibody, followed by another overnight incubation with ^125^I‐β‐endorphin. Samples were then incubated with goat anti‐rabbit IgG serum and normal rabbit serum, centrifuged, and the supernatant discarded prior to detection of bound ^125^I‐β‐endorphin in the remaining pellet with a gamma‐counter (PerkinElmer, Waltham, MA). A standard curve was generated from which the concentration of β‐endorphin present in each sample was extrapolated. The detection range of the kit used is 10–1280 pg/ml.

### 2.8 Disruption of MOR signaling

To determine whether MORs contribute differentially in male and female mice to ABA, MOR function was inhibited in two ways: first by genetic deletion of the MOR using knockout mice discussed above, and second by administration of the MOR antagonist naloxone hydrochloride to wild‐type animals (NAL, 5 mg/kg i.p., Sigma‐Aldrich, St. Louis, MO). NAL was administered twice daily at 0.5 hours until lights out and again 4.5 hours later. Saline‐treated control animals received two injections of 0.9% NaCl sterile saline solution (0.1 ml) at the same time. Animals were first habituated to i.p. injections of saline solution during baseline data collection (one injection per day). Unlike previous ABA experiments in which animals were sacrificed on a predetermined day, animals in these experiments were allowed to proceed through ABA uninterrupted until either 20% of initial bodyweight had been lost or 6 days had passed, at which point all animals were removed from the study. Upon completion of the experiment, animals were humanely euthanized.

### 2.9 Statistical analyses

Detailed information regarding specific statistical tests used is given in the results section. All data were analyzed using Prism (GraphPad Software Inc., San Diego, CA). Data are presented as mean ± SD. Differences were considered significant when *p* ≤ 0.05.

## 3 RESULTS

### 3.1 Activity‐based anorexia (ABA) can be reliably generated in wild‐type mice

One of the most widely used and well‐established animal models is activity‐based anorexia (ABA) as it closely mimics several key features of AN, including severely restricted feeding and excessive exercise (Klenotich & Dulawa, 2012). We first verified that we were able to reproduce the increased wheel running during the hours preceding food presentation known as food anticipatory activity (FAA) as well as bodyweight loss in response to restricted feeding in female and male wild‐type mice (Figure 1). Data from female mice are presented in panels a‐c. Data from male mice are presented in panels d‐f. Wheel revolutions per 15‐minute bin over the course of 3 days in ABA are first shown for either sex (Figure 1a,d). The daily cumulative total of wheel revolutions run during FAA, the four hours preceding food presentation, are shown in panels b (female) and e (male). As an ABA experiment progresses, the sample size inevitably gets smaller given that animals are removed from an ABA study when they lose 20% of their baseline bodyweight or greater. We have therefore elected to display the data up to the point that removal of animals from the experiment became necessary. For females, this is up to 4 days of ABA; for males, 3 days. In both sexes, we were able to reliably observe a significant increase in FAA in response to the ABA paradigm. In females, repeated measures one‐way ANOVA revealed a significant overall effect after 4 days of ABA: *F*_(1.784, 12.49)_ = 5.45, *p* = 0.0224 (Figure 1b, n = 8, 24 hr: 29 ± 40.48 mean ± SD, 48 h: 402.6 ± 709.1, 72 hr:2615 ± 3481, 96 hr: 5085 ± 4907). Repeated measures one‐way ANOVA revealed a significant overall effect in males after 3 days of ABA: *F*_(1.129, 5.646)_ = 7.575, *p* = 0.0334, n = 6, 24 hr: 202.3 ± 403.8, 48 hr: 1915 ± 2352, 72 hr: 6099 ± 5343 (Figure 1e). Bodyweight loss over the course of ABA is shown as the animal's daily bodyweight expressed as a percentage of its baseline average for both females (Figure 1c; repeated measures one‐way ANOVA; *F*_(1.309, 9.164)_ = 71.35, *p* < 0.0001, n = 8, 24 hr: 93.12%±1.954, 48 hr: 88.61%±1.494, 72 hr: 84.63% ± 2.474, 96 hr: 82.19%±3.315) and males (Figure 1f; repeated measures one‐way ANOVA; *F*_(1.559, 10.92)_ = 77.61, *p* < 0.0001, n = 8, 24 hr: 92.31% ± 2.694, 48 hr: 86.72% ± 3.278, 72 hr: 82.70% ± 3.464). Relative to the night before the initiation of ABA, a significant decrease in food intake was observed when hours of food access were restricted to the first two hours of the dark cycle in both females and males (Table 1).

**TABLE 1:** Food intake data expressed as a percentage of the animal's baseline average food consumption

Sex	Condition	Food consumed as percentage of baseline average (mean ± SD)				
		0 hr in ABA	24 hr in ABA	48 hr in ABA	72 hr in ABA	96 hr in ABA
Female	WT^a^	n = 8, 99.26%±15.31	n = 8, 23.43%±8.799	n = 8, 38.75%±7.506	n = 8, 40.94%±9.255	n = 8, 45.30%±6.763
Female	MOR ko^b^	–	n = 9, 15.48%±4.751	n = 9, 29.35%±8.339	n = 9, 32.88%±7.735	n = 9, 41.86%±10.35
Male	WT^c^	n = 7, 110%±4.793	n = 7, 17.83%±9.297	n = 8, 46.67%±13.26	n = 8, 39.34%±11.12	–
Male	MOR ko^d^	–	n = 13, 13.10%±3.653	n = 13, 33.99%±11.66	n = 13, 31.25%±11.72	–
Male	NAL^c^	–	n = 9, 18.70%±4.780	n = 8, 41.98%±10.69	n = 8, 43.49%±10.89	–

All data presented as mean ± SD. a) One‐way repeated measures ANOVA in WT females *p* < 0.001; *F*_(2.125, 14.88)_ =94.12. b) Two‐way repeated measures ANOVA comparing MOR knockout females to WT females followed post‐hoc analysis via Sidak's multiple comparisons. Sidak's multiple comparisons were not significant (24 hr WT vs. MOR ko:*p* = 0.1679; 48 hr WT vs. MOR ko: *p* = 0.1047, 72 hr WT vs. MOR ko: *p* = 0.2625; 96 hr WT vs. MOR ko: *p* = 0.8916) despite an overall effect (*p* = 0.149; *F*_(1,15)_ =7.565). c) One‐way repeated measures mixed effects model to account for missing datapoint due to data collection error in WT males; *p* < 0.001; *F*_(3,26)_ =105.5. d) Two‐way repeated measures mixed effects model comparing MOR knockout males to WT males followed by post‐hoc analysis via Sidak's multiple comparisons. Sidak's multiple comparisons were not significant (24 hr WT vs. MOR ko:*p* = 0.555; 48 hr WT vs. MOR ko: *p* = 0.1256, 72 hr WT vs. MOR ko: *p* = 0.3481) despite an overall effect (*p* = 0.003; *F*_(1,56)_ =9.647). e) Two‐way repeated measures mixed effects model; *p* = 0.9688; *F*_(1,42)_ =0.001543.

### 3.2 *Pomc* mRNA is transiently increased in both female and male animals undergoing ABA

**FIGURE 2:**
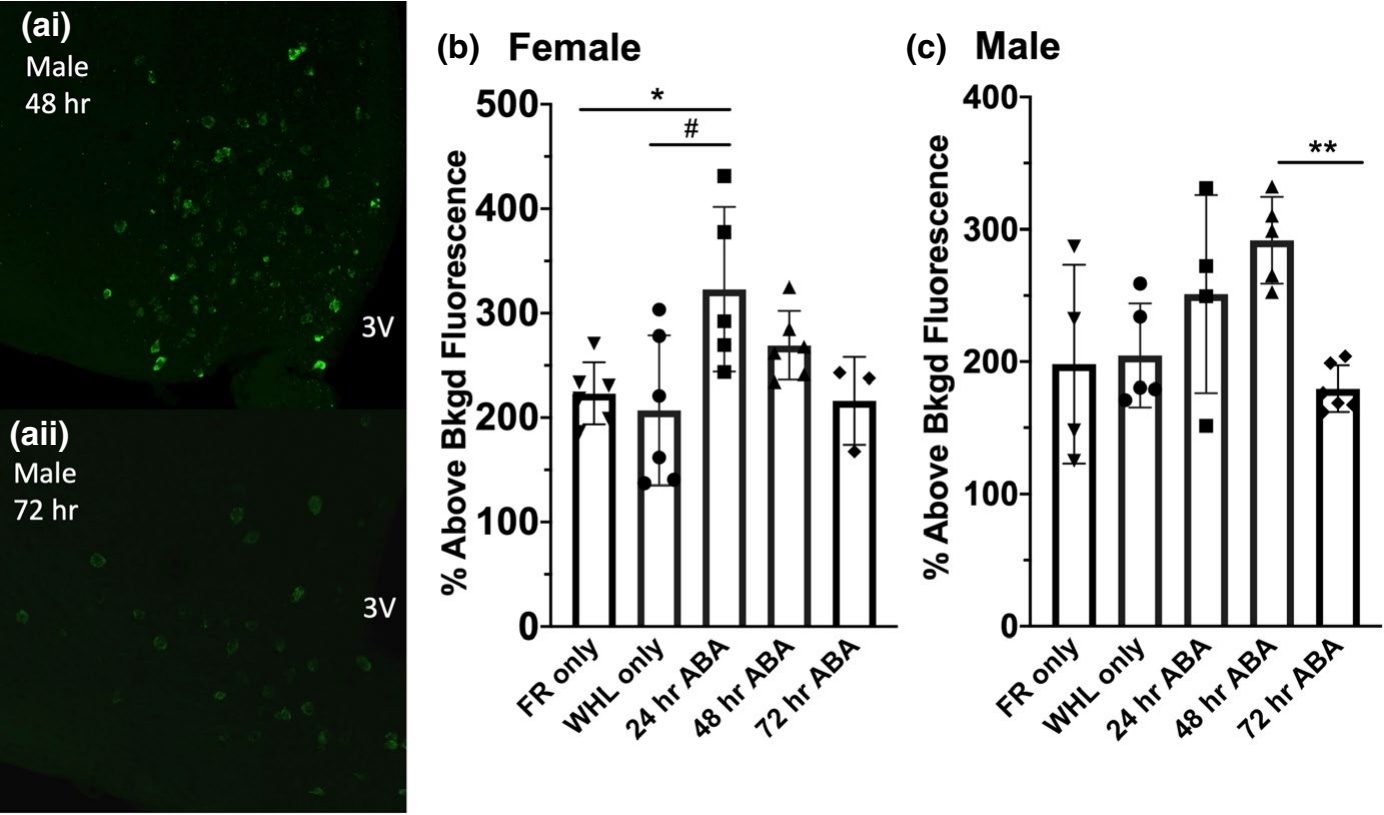
*Pomc* mRNA is transiently elevated in both female and male mice undergoing ABA. Representative confocal images taken at 40× of *Pomc* mRNA detected with Alexa488‐labeled probe in the arcuate nucleus of the hypothalamus from male animals sacrificed after 48 hr (ai) or 72 hours (aii) in ABA. Summary data from females are shown in panel b: *p = 0.0500, #*p* = 0.0177, one‐way ANOVA. Summary data from males are shown in panel c: *p = 0.0110, one‐way ANOVA. Summary data are presented as mean ± SD. Additional details are found in the results section. 3 V = third ventricle. FR = food restricted. WHL = wheel. n numbers were as follows: female: FR only: n = 6, WHL only: n = 6, 24 hr ABA: n = 5, 48 hr ABA: n = 6, 72 hr ABA: n = 3, male: FR only: n = 4, WHL only: n = 5, 24 hr ABA: n = 5, 48 hr ABA: n = 5, 72 hr ABA: n = 6

*Pomc* mRNA levels change in response to an organism's energy state such that during times of positive energy balance *Pomc* mRNA is increased (Schwartz et al., 1997) and during times of negative energy balance *Pomc* mRNA is decreased (Benoit et al., 2002; Mizuno et al. 1998). Paradoxically, Hillebrand and colleagues have previously shown a transient increase in *Pomc* mRNA levels in female rats undergoing ABA despite the animal existing in a state of negative energy balance (Hillebrand et al., 2006). We performed fluorescent *in situ* hybridization to detect changes in *Pomc* mRNA levels in female and male wild‐type mice having undergone one, two, or three days of ABA or a control condition (food restriction only or wheel running only). Example images are shown in Figure 2a. The fluorescent intensity of each *Pomc*‐expressing cell was expressed as a percentage of background fluorescence intensity specific to that image and an overall average of fluorescent intensity above background was determined for each animal for statistical analysis. One‐way ANOVA revealed a significant difference in means between treatment groups (Figure 2b, *F*_(4,21)_ = 3.892, *p* = 0.0162). After one day of ABA, female mice showed a significant increase in *Pomc* mRNA fluorescent intensity compared to both food restricted controls (Figure 2b, Tukey's multiple comparison, *p = 0.0500, FR only: n = 6, 223.2%±29.75, 24 h ABA: n = 5, 322.9%±78.83) and wheel running only controls (Figure 2b, Tukey's multiple comparison, #*p* = 0.0177, WHL only: n = 6, 207.1%±71.95, 24 hr ABA: n = 5, 322.9%±78.83). Female mice showed a peak in fluorescent intensity after one day of ABA. By thee days of ABA, fluorescent intensity values had essentially returned to levels observed in either control condition (Figure 2b, Tukey's multiple comparison, FR only vs. 72 hr ABA: *p* = 0.9997, FR only: n = 6, 223.2% ± 29.75, 72 hr ABA: n = 3, 216.2% ± 42.06, WHL only vs. 72 hr ABA: *p* = 0.9993, WHL only: n = 6, 207.1% ± 71.95, 72 hr ABA: n = 3, 216.2% ± 42.06).

As observed in the female mice, a significant difference in mean fluorescent intensity was observed between treatment groups in male mice (Figure 2c, one‐way ANOVA, *F*_(4,19)_ =4.398, *p = 0.0110). Unlike in females where the peak fluorescent intensity was observed after one day of ABA (Figure 2b, 24 hr ABA: n = 5, 322.9%±78.83), in male animals the peak fluorescent intensity was observed after two days of ABA (Figure 2c, 48 hr ABA: n = 5, 291.9%±32.71). Fluorescent intensity was significantly decreased by three days of ABA compared to two days of ABA (Figure 2c, Tukey's multiple comparison, 72 hr ABA vs. 48 hr ABA: **p = 0.0098, 72 h ABA: n = 6, 179.6%±17.66, 48 h ABA: n = 5, 291.9% ± 32.71). No significant difference was observed between either control condition and thee days of ABA (Figure 2c, Tukey's multiple comparison, FR only vs. 72 hr ABA: *p* = 0.9756, FR only: n = 4, 198.1 ± 75.10, 72 hr ABA: n = 6, 179.6%±17.66, WHL only vs. 72 hr ABA: *p* = 0.9130, WHL only: n = 5, 204.7%±39.34, 72 hr ABA: n = 6, 179.6%±17.66).

### 3.3 Peripheral levels of β‐endorphin increase over the course of ABA

**FIGURE 3:**
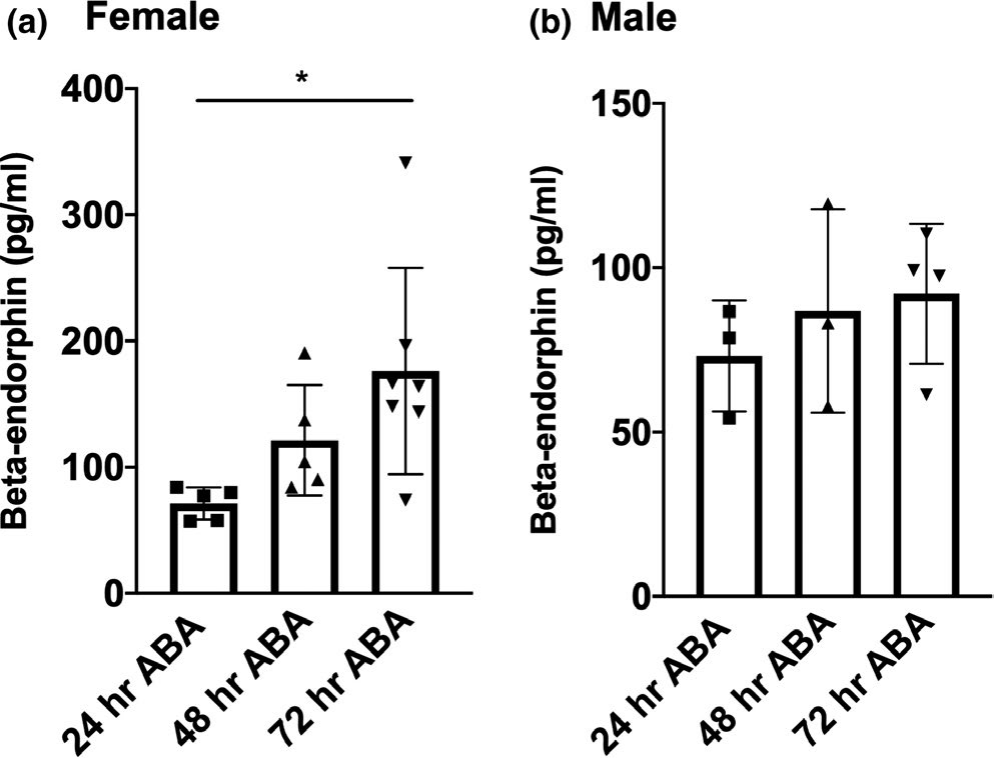
Peripheral levels of β‐endorphin are elevated in response to ABA. Radioimmunoassay was used to detect circulating levels of beta‐endorphin in peripheral blood collected from female (a) and male (b) after varying days of exposure to ABA. Data are presented as mean ± SD. *p = 0.0223. Data were analyzed using one‐way ANOVA. Additional details are found in the results section. n numbers were as follows: female: 24 hr ABA: n = 5, 48 hr ABA: n = 5, 72 hr ABA: n = 7, male: 24 hr ABA: n = 3, 48 h ABA: n = 3, 72 hr ABA: n = 4

To determine whether the increase in *Pomc* mRNA observed might lead to an increase in circulating levels of β‐endorphin, we performed radioimmunoassays to detect levels of β‐endorphin in serum from animals euthanized after varying days in ABA. One‐way ANOVA revealed a statistically significant effect in female mice (Figure 3a, *F*_(2,14)_ =4.701, *p* = 0.0274). Specifically, a significant increase in β‐endorphin was observed when female mice completed 3 days of ABA compared to 1 day (Figure 3a, Tukey's multiple comparison, *p* = 0.0223, 24 hr ABA: n = 5, 71.25 ± 12.82, 72 hr ABA: n = 7, 176.2 ± 81.78). No significant difference was found between one and two days in ABA (Figure 3a, Tukey's multiple comparison, *p* = 0.3940, 24 hr ABA: n = 5, 71.25 ± 12.82, 48 hr ABA: n = 5, 121.4 ± 43.79) or two and three days in ABA (Figure 3a, Tukey's multiple comparison, *p* = 0.2817, 48 hr ABA: n = 5, 121.4 ± 43.79, 72 hr ABA: n = 7, 176.2 ± 81.78). Mean concentration of β‐endorphin from male mice is presented in Figure 3b, though statistical analysis was not performed due to the low number of samples from male mice that met quality standards (Figure 3b, 24 hr ABA: n = 3, 73.18 ± 16.92, 48 hr ABA: n = 3, 86.87 ± 30.99, 72 hr ABA: n = 4, 92.08 ± 21.24).

### 3.4 Food anticipatory activity (FAA) is blunted after MOR deletion in both male and female mice

**FIGURE 4:**
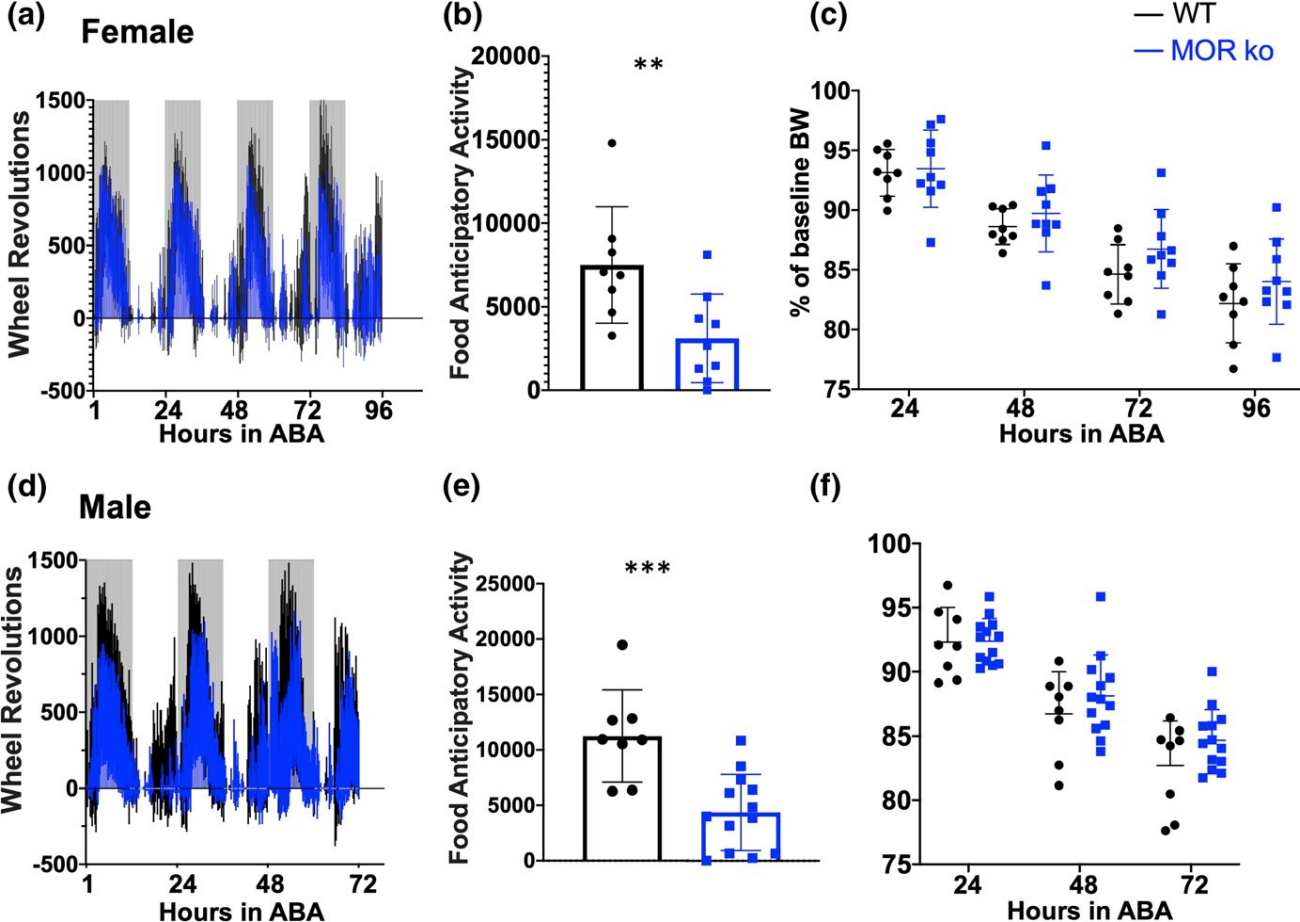
Both male and female MOR knockout mice display blunted FAA. Wheel revolutions per 15‐minute time bin over the course of 3 days in ABA are shown for female (a) and male (d) mice. Gray bars denote the dark cycle. Highest daily total of FAA for each animal is shown in panels b (female: WT: n = 8, MOR ko: n = 9, **p = 0.0099, unpaired two‐tailed T test) and e (male: WT: n = 8, MOR ko: n = 13, ***p = 0.0006, unpaired two‐tailed T test). Daily bodyweight expressed as a percentage of the animal's baseline average are shown in panels c and F for females and males, respectively. Summary data presented as mean± SD. Data shown in panels c and f were analyzed repeated measures two‐way ANOVA. Additional details are found in the results section

After detecting elevations in *Pomc* mRNA and β‐endorphin concentration in response to ABA, we next investigated whether MOR activation contributes to the development of ABA. Previous studies have shown that FAA can be blunted in female MOR knockout mice undergoing ABA (Kas et al., 2004); given the known sex differences in AN and ABA however, we hypothesized that MOR activation may contribute differentially to the development of ABA in males versus females. Data from female and male MOR knockout mice are shown in Figure 4. The overall pattern of wheel revolutions per 15‐minute bin over the course of 3 days in ABA is shown for female (Figure 4a) and male (Figure 4d) mice (wild‐type animals in black, MOR knockout animals in blue). There is day‐to‐day variability in FAA for any given animal and a distribution in days needed to lose 20% of initial bodyweight, and thus, FAA is presented for each animal at the day that wheel revolutions were highest for the individual. In the majority of instances, the highest level of FAA is displayed on the same day that the animal reaches the experimental endpoint. In instances where this was not the case, animals appeared to be exhausted and displaying minimal engagement with the running wheel. Both female (Figure 4b) and male (Figure 4e) MOR knockout mice display significant reductions in FAA compared to wild type; female: unpaired, two‐tailed T test, **p = 0.0099, WT: n = 8, 7500 ± 3488, MOR knockout: n = 9, 3103 ± 2644; male: unpaired, two‐tailed T test, ***p = 0.0006, WT: n = 8, 11247 ± 4166, MOR knockout: 4363 ± 3424. No significant differences in bodyweight loss were detected between MOR knockout animals and wild types in both females (Figure 4c; two‐way repeated measures ANOVA; *F*_(1,15)_ = 1.12, *p* = 0.3066, WT: 24 hr ABA: 93.12% ± 1.954, 48 hr ABA: 88.61% ± 1.494, 72 hr ABA: 84.63% ± 2.474, 96 hr ABA: 82.19 ± 3.315, MOR knockout: 24 hr ABA: 93.45% ± 3.214, 48 hr ABA: 89.72% ± 3.199, 72 hr ABA: 86.75% ± 3.304, 84.00% ± 3.555) and males (Figure 4f; two‐way repeated measures ANOVA; *F*_(1,19)_ = 1.014, *p* = 0.3267, WT: 24 hr ABA: 92.31% ± 2.694, 48 hr ABA: 86.72% ± 3.278, 72 hr ABA: 82.70% ± 3.464, MOR knockout: 24 hr ABA: 92.38% ± 1.744, 48 hr ABA: 88.13% ± 3.178, 72 hr ABA: 84.68% ± 2.369). We did not detect significant differences in food consumption at any specific timepoint in ABA between MOR knockouts and WTs of either sex (Table 1).

### 3.5 MOR antagonism reduces FAA only in male mice

**FIGURE 5:**
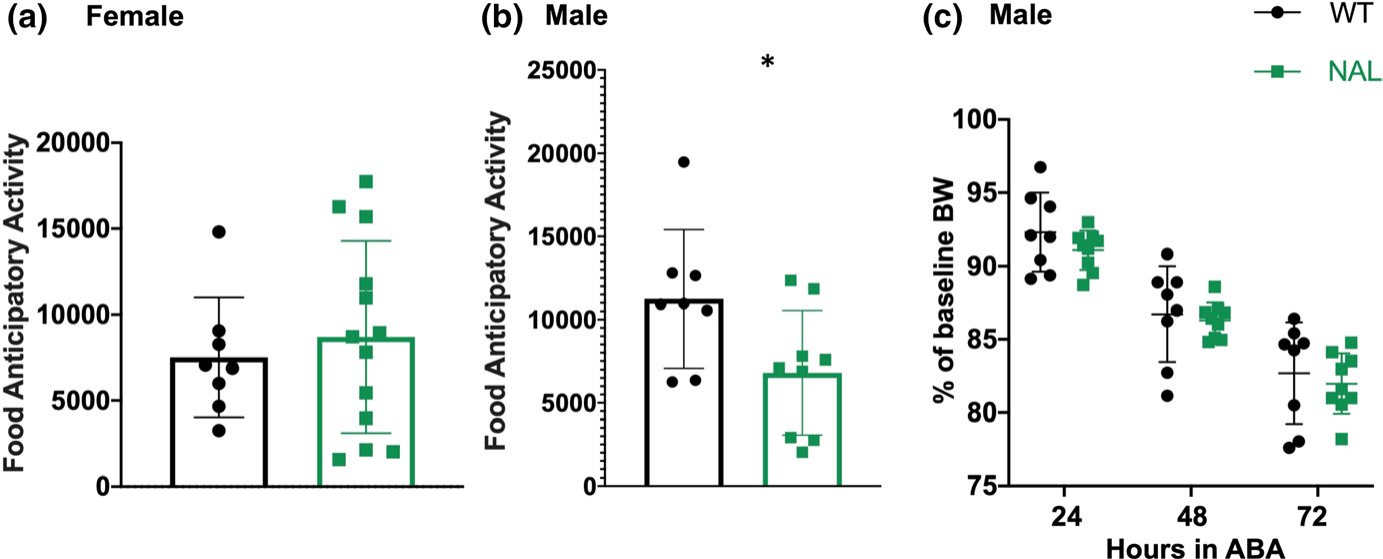
Male, but not female, mice display reduced FAA in response to naloxone treatment. Highest daily FAA totals are shown for female (a, n = 13) and male (b, n = 9) mice undergoing ABA. Daily bodyweight expressed as a percentage of baseline average are shown for male mice (c). Control mice are shown in black; mice treated with naloxone (NAL) are shown in green. Summary data presented as mean ± SD. In panel b, *p = 0.0352. Unpaired two‐tailed T tests were used to analyze data shown in panels a and b; repeated measures two‐way ANOVA was used to analyze data shown in panel c. Additional details are found in the results section

We elected to further investigate the hypothesis that MOR activation contributes to the development of ABA by treating wild‐type animals undergoing ABA with the MOR antagonist naloxone hydrochloride (NAL, 5 mg/kg). Here, we observed a sex‐specific effect with males but not females show showing a significant reduction in FAA in response to NAL treatment (Figure 5a: Female, unpaired T test, two‐tailed, *p* = 0.5951, WT: n = 8, 7500 ± 3488, NAL: n = 13, 8695 ± 5588; Figure 5b: Male, unpaired T test, two‐tailed, *p = 0.0352, WT: n = 8, 11247 ± 4166, NAL: n = 9, 6809 ± 3744). Despite the reduction in FAA observed in males administered NAL, there was not a significant change in bodyweight in response to NAL treatment (Figure 5c; two‐way repeated measures ANOVA; *F*_(1,15)_ = 0.5425, *p* = 0.4728, WT: 24 hr ABA: 92.31%±2.694, 48 h ABA: 86.72%±3.278, 72 hr ABA: 82.70%±3.464, NAL: 24 hr ABA: 91.08%±1.344, 48 hr ABA: 86.30%±1.219, 72 hr ABA: 81.97%±2.066). There was also no significant difference in food intake in mice receiving NAL compared to WT (Table 1).

## 4 DISCUSSION

In the current study, we found that *Pomc* mRNA levels were transiently elevated in both female and male mice undergoing ABA and that there was also a concomitant rise in circulating β‐endorphin. A role for β‐endorphin action in FAA was indicated in studies in mice lacking MORs and studies where MOR function was pharmacologically antagonized. While previous work had indicated that deletion of MORs decreased FAA in female mice, the present study extends the findings to males as well. Interestingly, administration of the MOR antagonist naloxone hydrochloride (NAL) reduced FAA in male, but not female, mice. We did not observe changes in bodyweight or food intake in response to MOR deletion or NAL administration. The results presented here suggest (1) that increased *Pomc* transcription is transient and observed in both female and male mice, (2) targeted inhibition of β‐endorphin function via MOR genetic deletion selectively blunts FAA in female and male animals, yet (3) only male mice show a similar blunting of FAA in response to NAL treatment. These results highlight the potential of sex‐specific differences in the mechanisms underlying ABA and highlight the potential need for sex‐specific treatments in individuals with AN.

### 4.1 Sex differences

Many epidemiological studies have shown eating disorders in general, and especially AN, to be much more common in females compared to males (Keski‐Rahkonen & Mustelin, 2016; Smink et al., 2012). Given the unequal sex distribution observed in the human population, many previous rodent ABA studies were only conducted on female animals (Hillebrand, Kas, et al., 2005; Hillebrand, Kas, et al., 2005; Hillebrand et al., ,,,^2005^, ^2006^; Kas et al., 2004). However, processes underlying energy homeostasis, and more specifically the physiology of feeding behaviors, are known to be highly sexually dimorphic (Asarian & Geary, 2013). Moreover, a considerable degree of evidence suggests that POMC neurons are sexually dimorphic as well: Female mice have significantly more POMC neurons compared to male mice and electrophysiological recordings from POMC neurons of female mice displayed an increase in firing rate as well as a decrease in resting membrane potential (Wang et al., 2018); male, but not female, mice from which the vesicular glutamate transporter *Vglut2* is deleted from POMC neurons are unable to maintain normal bodyweight (Dennison et al., 2016); similarly, female mice lacking a G protein‐coupled receptor (Gpr17) from POMC neurons better maintained energy homeostasis relative to males (Reilly et al., 2019). We therefore felt it necessary to revisit experiments performed only in female rodents to determine whether increases in *Pomc* mRNA and decreased FAA in MOR knockout mice are also true in males. We show for the first time in male mice a transient increase in *Pomc* mRNA and confirm the previously reported observation of a similar transient increase in *Pomc* mRNA in female mice (Hillebrand et al., 2006). Interestingly, we observed a temporal difference in the peak of the increase in *Pomc* transcription, with males showing a peak in fluorescence on day two compared to day one in females.

### 4.2 Wheel running and opioid‐mediated reward

Wheel running is considered rewarding in rodents as they display conditioned place preference for a particular side of a chamber where access is given to a running wheel (Lett et al., 2001). Moreover, engagement in voluntary wheel running has been shown to reduce the consumption of other known rewarding stimuli such as a high‐fat diet (Liang et al., 2015) and various drugs of abuse including heroin (Smith & Pitts, 2012) and cocaine (Cosgrove et al., 2002). As an endogenous opioid, β‐endorphin is a critical mediator of reward and it has been shown that pharmacological blockade of this system can reduce wheel running (Rasmussen & Hillman, 2011). In ABA, animals will run on the running wheel to the point of exhaustion and death if the investigator does not intervene; we therefore hypothesized that the animals consider wheel running rewarding and that β‐endorphin concentrations are increased during ABA to signal this reward. The observed increase in β‐endorphin after three days in ABA compared to one day supports this hypothesis and might explain why rodents choose to engage in wheel running despite limited resources (restricted feeding). These findings are in line with previous work that showed that leptin signaling through the ventral tegmental area, a key brain region in reward processing, can reduce hyperactivity observed in ABA (Verhagen et al., 2011). We elected to measure circulating levels of β‐endorphin due to being unable to collect an adequate volume of cerebrospinal fluid (CSF) to allow for reliable peptide detection by radioimmunoassay. We acknowledge the pitfalls of using this approach given that positive and negative correlations between central and peripheral levels of β‐endorphin have been reported in rodents and humans (Aravich et al., 1993; Baker et al., 1997; Kosten et al., 1987; Martinez et al., 1993; Vecsei et al., 1992; Yamamoto et al., 2000). Despite the caveat that peripheral β‐endorphin levels do not always reflect central β‐endorphin levels, we elected to determine whether our hypothesis might be supported. A second limitation of the current study is the use of a global rather than site‐specific MOR knockout. While we are unable in the current study to point toward specific brain sites as likely candidate regions underlying ABA, future studies should address this question using site‐specific MOR deletion.

### 4.3 Food intake and bodyweight

In addition to playing an important role in the signaling of rewarding stimuli, β‐endorphin has also been shown to influence feeding behavior, albeit in a complex manner given that opposing effects have been observed and given the fact that β‐endorphin contributes to both homeostatic and hedonic aspects of food intake (Nogueiras et al., 2012). Exogenous intracerebroventricular administration of β‐endorphin has been shown to increase food intake (Silva et al., 2001), yet genetic deletion of β‐endorphin also leads to increased food intake and bodyweight in male mice (Appleyard et al., 2003; Low et al., 2003). Given the complex nature of the role of β‐endorphin in feeding behavior, perhaps it is not surprising that we did not observe effects on bodyweight and food intake when inhibiting the actions of β‐endorphin as the predicted outcome is not clear. Moreover, while we initially hypothesized that the melanocortin system was uniformly involved in all features of ABA, that is, equal effect on wheel running behavior as well as feeding behavior and resulting weight loss, the results presented in the current study seem to suggest that β‐endorphin specifically regulates the wheel running feature of ABA, leaving open the possibility for a second neuronal system to regulate the feeding and bodyweight changes observed in ABA. A potential candidate system could be the agouti‐related peptide (AGRP) neurons, residing in the arcuate nucleus of the hypothalamus alongside POMC neurons, that potently stimulate food intake (Aponte et al., 2011); indeed, exogenous administration of AGRP to rodents undergoing ABA shows greater food intake relative to controls (Hillebrand et al., 2006; Kas et al., 2003). Exactly how these two neuronal populations with opposing effects on feeding behavior could work in concert to contribute to the development of ABA remains to be determined, however.

## 5 CONCLUSIONS

Finally, while many findings in the current study were similar between female and male mice (both sexes displaying elevations in *Pomc* transcription, elevated β‐endorphin concentration, and blunted FAA in response to MOR deletion), only male mice showed reduced FAA when treated with NAL. This could suggest two things: first is the possibility that there are sex‐specific differences in the degree of involvement of the opioidergic system in ABA, highlighting the utility and necessity of using both sexes when conducting ABA studies; and second is the potential need for sex‐specific approaches to treatment in individuals with AN. Indeed, while not fully understood, men and women show differential responses to opioidergic drugs (Bartley & Fillingim, 2013) and it is possible that the NAL dose used in the current study, a mid‐range dose shown in the literature to be effective in rodent wheel running studies (Sisti & Lewis, 2001) was too low to see the effect observed in males. The potential need for sex‐specific approaches to AN treatment has been indicated by emerging evidence, suggesting that AN in males has a different clinical presentation when compared to females (Coelho et al., 2018). Eating disorders in males have been described as “under‐diagnosed, undertreated, and misunderstood” (Strother et al., 2012). It is therefore imperative that potential sex differences be identified and studied further in both ABA and AN studies.

## CONFLICT OF INTERESTS

The authors declare no competing financial interests.

## AUTHOR CONTRIBUTIONS

CMD performed the conception and design of work, data acquisition, analysis, and interpretation, and wrote the manuscript. STH involved in conception and design of work, and data analysis and interpretation, and wrote manuscript. All authors approved the final version of the manuscript and agree to be accountable for all aspects of the work in ensuring that questions related to the accuracy or integrity of any part of the work are appropriately investigated and resolved. All persons designated as authors qualify for authorship, and all those who quality for authorship are listed.
